# Water Extract from Inflorescences of Industrial Hemp Futura 75 Variety as a Source of Anti-Inflammatory, Anti-Proliferative and Antimycotic Agents: Results from In Silico, In Vitro and Ex Vivo Studies

**DOI:** 10.3390/antiox9050437

**Published:** 2020-05-17

**Authors:** Giustino Orlando, Lucia Recinella, Annalisa Chiavaroli, Luigi Brunetti, Sheila Leone, Simone Carradori, Simonetta Di Simone, Maria Chiara Ciferri, Gokhan Zengin, Gunes Ak, Hassan H. Abdullah, Estefanía Cordisco, Maximiliano Sortino, Laura Svetaz, Matteo Politi, Paola Angelini, Stefano Covino, Roberto Venanzoni, Stefania Cesa, Luigi Menghini, Claudio Ferrante

**Affiliations:** 1 Department of Pharmacy, University “G. d’Annunzio” of Chieti-Pescara, 66100 Chieti, Italy; giustino.orlando@unich.it (G.O.); lucia.recinella@unich.it (L.R.); annalisa.chiavaroli@unich.it (A.C.); luigi.brunetti@unich.it (L.B.); sheila.leone@unich.it (S.L.); simone.carradori@unich.it (S.C.); disimonesimonetta@gmail.com (S.D.S.); Mariachiara.ciferri@outlook.it (M.C.C.); matteo.politi@unich.it (M.P.); claudio.ferrante@unich.it (C.F.); 2 Department of Biology, Science Faculty, Selcuk Universtiy, Campus, Konya, Konya 42130, Turkey; gokhanzengin@selcuk.edu.tr (G.Z.); akguneselcuk@gmail.com (G.A.); 3 Chemistry Department, College of Education, Salahaddin University-Erbil, Erbil 44001, Iraq; hassan.abdallah@su.edu.krd; 4 School of Pharmaceutical Sciences, Universiti Sains Malaysia, USM, Penang 11800, Malaysia; 5 Farmacognosia, Facultad de Ciencias Bioquímicas y Farmacéuticas, Universidad Nacional de Rosario, Suipacha 531, Rosario 2000, Argentina; ecordisco@fbioyf.unr.edu.ar (E.C.); msortino@fbioyf.unr.edu.ar (M.S.); lsvetaz@fbioyf.unr.edu.ar (L.S.); 6 Centro de Referencia de Micología, Facultad de Ciencias Bioquímicas y Farmacéuticas, Universidad Nacional de Rosario, Suipacha 531, Rosario 2000, Argentina; 7 Department of Chemistry, Biology and Biotechnology, University of Perugia, 06100 Perugia, Italy; stefano.covino@unipg.it (S.C.); roberto.venanzoni@unipg.it (R.V.); 8 Department of Drug Chemistry and Technologies, “Sapienza” University of Rome, 00185 Rome, Italy

**Keywords:** futura 75, serotonin, dopamine, kynurenine, antimycotic, anti-proliferative

## Abstract

Industrial hemp (*Cannabis sativa*) is traditionally cultivated as a valuable source of fibers and nutrients. Multiple studies also demonstrated antimicrobial, anti-proliferative, phytotoxic and insecticide effects of the essential oil from hemp female inflorescences. On the other side, only a few studies explored the potential pharmacological application of polar extracts from inflorescences. In the present study, we investigated the water extract from inflorescences of industrial hemp Futura 75 variety, from phytochemical and pharmacological point of view. The water extract was assayed for phenolic compound content, radical scavenger/reducing, chelating and anti-tyrosinase effects. Through an ex vivo model of toxicity induced by lipopolysaccharide (LPS) on isolated rat colon and liver, we explored the extract effects on serotonin, dopamine and kynurenine pathways and the production of prostaglandin (PG)E_2_. Anti-proliferative effects were also evaluated against human colon cancer HCT116 cell line. Additionally, antimycotic effects were investigated against *Trichophyton rubrum, Trichophyton interdigitale, Microsporum gypseum.* Finally, in silico studies, including bioinformatics, network pharmacology and docking approaches were conducted in order to predict the putative targets underlying the observed pharmacological and microbiological effects. Futura 75 water extract was able to blunt LPS-induced reduction of serotonin and increase of dopamine and kynurenine turnover, in rat colon. Additionally, the reduction of PGE_2_ levels was observed in both colon and liver specimens, as well. The extract inhibited the HCT116 cell viability, the growth of *T. rubrum* and *T. interdigitale* and the activity of tyrosinase, in vitro, whereas in silico studies highlighting the inhibitions of cyclooxygenase-1 (induced by carvacrol), carbonic anhydrase IX (induced by chlorogenic acid and gallic acid) and lanosterol 14-α-demethylase (induced by rutin) further support the observed pharmacological and antimycotic effects. The present findings suggest female inflorescences from industrial hemp as high quality by-products, thus representing promising sources of nutraceuticals and cosmeceuticals against inflammatory and infectious diseases.

## 1. Introduction

Industrial hemp (*Cannabis sativa*) has been long extensively cultivated throughout history as a valuable source of fibers and nutrients [1]. Specifically, the fibers, isolated from the stalk, are used for manufacturing ropes, paper, clothing and construction materials (thermal and acoustic insulation) [2], whereas hemp seeds demonstrated a high nutritional value, due to their richness in minerals, vitamins (i.e., A, C and E complexes), carbohydrates, proteins and lipids, these last consisting of linoleic (ω-6) and α-linolenic acid (ω-3) in the ideal ratio 3:1 [3]. Additionally, industrial hemp varieties are also bred to produce tetrahydrocannabinol (THC) in traces (<0.3%) [4], and only certified varieties, with THC content < 0.2%, are admitted to cultivation, according to National and International Regulations (EU Regulation N°. 1124/2008-12 November 2008; Italian Regulation n°172/2017). Although the hemp chain production was principally focused on fiber production and seed-deriving foods, in the last years there has been a renewed interest in the study and valorization of hemp-deriving extracts, that are sources of terpenes, terpenophenolics, amino acids, fatty acids, sugars, hydrocarbons, flavonoids, with promising health-promoting and pharmacological effects [5]. In this regard, multiple studies focused on the antimicrobial, anti-proliferative, phytotoxic and insecticide effects of the essential oil from hemp female inflorescences [6,7,8], sold dried for technical use and usually considered as a waste material of industrial hemp chain production [9]. On the other side, only a few studies explored the potential pharmacological application of polar extracts from inflorescences [10,11]. In our previous study, we showed antioxidant/anti-inflammatory and antimycotic properties of the certified hemp variety “Futura 75” [10], whose essential oil from inflorescences was also reported as an antibacterial and anti-proliferative agent [8]. In the present study, we aimed to further characterize the water extract from the inflorescences of this hemp variety, through a qualitative phytochemical analysis and a pharmacological investigation aimed to evaluate protective effects in a toxicological experimental paradigm constituted by isolated rat colon and liver exposed to lipopolysaccharide (LPS). In this regard, we explored the effects of water hemp extract on serotonin, dopamine and kynurenine pathways, in rat colon, whereas prostaglandin (PG)E_2_ levels were measured in both colon and liver. Anti-proliferative effects were also assayed against the human colon cancer HCT116 cell line. Taking into consideration the inhibitory effects induced by this extract on both *Candida albicans* and *C. tropicalis* [10], we also explored the antimycotic effects of Futura 75 water extract on multiple dermatophytes species, namely *Trichophyton rubrum, Trichophyton interdigital, Microsporum gypseum.* In parallel, we assayed the enzyme inhibition effect of the extract against tyrosinase, whose increased activity is related to skin hyperpigmentation, following mycotic infectious diseases, as well [12]. Finally, in silico studies, including bioinformatics, network pharmacology and docking approaches were conducted in order to predict the putative targets underlying the observed pharmacological and microbiological effects.

## 2. Materials and Methods 

### 2.1. Hemp Sample, Reagents and Standard Solutions

The plant material consists in female flowers of *Cannabis sativa* L cultivar ‘Futura 75′, ‘cultivated in Abruzzo Region (Italy). Female inflorescences were manually harvested from plant at full blooming state. Plant material was dried in ventilated oven (40 °C), soon after harvesting. Plant identity was confirmed botanically and morphologically by co-author Prof. Luigi Menghini, Associate Professor of Botany at the Department of Pharmacy, “G. d’Annunzio” University, Chieti (Italy).

Samples were kindly supplied by Hemp Farm Italia scarl (Tortoreto (TE), IT) during cultivation season 2017. 

### 2.2. Extract Preparation

Dried cultivar samples (0.2 g) were homogenized using a T25 digital Ultra-Turrax device for 30 s at 10,000 rpm. Subsequently, ultrasound-assisted extraction of the homogenate was carried out through a Trans-sonic T460 ultrasonic for 10 min. at 60 °C and full power (35 kHz). The obtained extract (solid–liquid ratio 1:50) was immediately centrifuged at 4000 rpm and 4 °C for 5 min, and finally dried through freeze-drying.

### 2.3. Phytochemical Analysis

Phenols and flavonoids levels were determined spectrophotometrically and the results were expressed as gallic acid equivalents (mg GAE/g extract) and rutin equivalents (mg RE/g extract), respectively. Antioxidant effects were investigated by radical scavenging 2,2-diphenyl-1-picrylhydrazyl (DPPH) and 2,2′-azino-bis(3-ethylbenzothiazoline-6-sulfonic acid) (ABTS) and reducing power (CUPRAC and FRAP) assays. Antioxidant results were expressed as trolox equivalents (TE). The enzyme inhibition towards tyrosinase was conducted with colorimetric assay, as well, and expressed as kojic acid equivalents. The experimental procedures for all these assays were comprehensively described in our previous studies [13,14]. 

Protonic magnetic resonance (^1^H-NMR) analysis was conducted through a Variant 300 MHz spectrometer using standard proton pulse sequence (s2pul) with pre-saturation. Sample was prepared by adding 60 µL of D_2_O as internal lock to 600 µL of the sample. Pre-saturation was carried out with relaxation delay 1 sec, pulse 45.0 degrees, acquisition time 1.706 sec, width 4803.1 Hz and 64 number of scans; partial suppression of the water solvent signals around 4.80 ppm was achieved.

### 2.4. Cell Culture

The anti-proliferative effect of industrial hemp extracts was studied using the human colon cancer HCT116 (ATCC^®^ CCL-247™) cell line, whose culturing conditions were detailed in our previous study [10]. The 3-(4,5-dimethylthiazol-2-yl)-2,5-diphenyltetrazolium bromide (MTT) test was employed for evaluating the basal cytotoxicity induced by the extract (10–500 µg/mL), in the 24 h following stimulation. The effect of the extract (500 µg/mL) on HCT116 cell spontaneous migration was also assayed through the wound healing experimental paradigm. The specifications about MTT and wound healing paradigms are reported in our previous paper [10].

### 2.5. Ex Vivo Model of LPS-Induced Toxicity in Isolated Rat and Liver Tissues

Male adult Sprague-Dawley rats (200–250 g) were sacrificed by CO_2_ inhalation (100% CO_2_ at a flow rate of 20% of the chamber volume per min) and liver and colon specimens were immediately collected and maintained in humidified incubator with 5% CO_2_ at 37 °C for 4 h, in DMEM buffer with added bacterial LPS (10 µg/mL) (incubation period). 

During the incubation period, colon and liver specimens were stimulated with water hemp extract (50–500 μg/mL). Tissue supernatants were collected, and the PGE_2_ level (ng/mg wet tissue) was measured by radioimmunoassay (RIA), as previously reported [15]. 

On the other hand, individual colon specimens were dissected and homogenized in perchloric acid solution (50 mM) for extracting serotonin (5-HT), 5-hydroxyindolacetic acid (5HIIA), dopamine (DA), dihydroxyphenylacetic acid (DOPAC), 3-hydroxykinurenine (3-HK) and kynurenic acid (KA) levels (ng/mg wet tissue). Briefly, colon 3-HK, 5-HT, 5HIIA, DA and DOPAC levels were analyzed through high performance liquid chromatography (HPLC) coupled to coulometric detection (reducing potential: −150 mV; oxidative potential: +300 mV), whereas the KA was determined through HPLC-fluorimetric analysis (excitation: 344 nm; emission: 398 nm) [16,17]

### 2.6. Antifungal Activity of the Extract

Microdilution techniques were conducted according to the guidelines M38-Ed3 of CLSI [18]. Tested extract was diluted with RPMI-1640 buffer, to final concentrations of 31.3–1000 µg/mL. *Trichophyton rubrum* (CCC 134–2000), *Trichophyton interdigitale* (CCC 202–2000) and *Microsporum gypseum* (CCC 115) were kindly provided by Reference Center of Mycology (CEREMIC), Faculty of Biochemical and Pharmaceutical Sciences, Rosario, Argentina. A (1.104 CFU/mL) inoculum suspension (100 µL) was added to each well (final volume in the well = 200 µL). A growth control-well (GCW) (containing medium, inoculum and the same amount of DMSO used in CTW, but compound-free) and a sterility control well (SCW) (sample, medium and sterile water instead of inoculum) were included. Samples were incubated at 28 °C for 72 h. Minimum Inhibitory Concentration (MIC) was defined as the lowest concentration giving 100% of inhibition. MIC ≤ 1000 µg/mL was considered as an index of antimycotic activity (no fungi growth observed). 

### 2.7. Bioinformatics and Docking Studies

The bioinformatics analysis was conducted according to the protocol described by Gu and colleagues [19]. The chemical structures were prepared with ChemSketch software and the related canonical SMILES were then processed by the SwissTargetPrediction and SwissADME platforms, for predicting putative targets and pharmacokinetic profile, respectively (http://www.swissadme.ch/index.php). The identification of predicted targets was confirmed through the use of UniProt database (https://www.uniprot.org/). Cytoscape software (3.7.2 version) was employed for the subsequent network pharmacology analysis.

### 2.8. Docking Calculations

Four compounds were picked for the docking calculations, namely, chlorogenic acid, gallic acid, carvacrol and rutin. Chlorogenic acid and gallic acid were docked against carbonic anhydrase IX enzyme, carvacrol against cyclooxygenase-1 enzyme and rutin against lanosterol 14-α-demethylase enzyme. The starting structures of the four compounds were first downloaded from ChemSpider online data base (http://www.chemspider.com) then optimized to the ground state structure using AM1 semiempirical method implemented in VegaZZ software [20]. On the other hand, the crystal structures of the proteins were downloaded from Protein Data Bank (PDB). PDBID: 3IAI was used to get the crystal structure of the carbonic anhydrase IX enzyme; PDBID: 4O1Z was used to get the crystal structure of the cyclooxygenase-1 enzyme; PDBID: 5TZL was used to get the crystal structure of the lanosterol 14-α-demethylase enzyme. In order to prepare the proteins for the docking simulation, all the water molecules and the co-crystalized heteromolecules were removed first followed by adding hydrogen atoms and neutralized using Kollman united atom charges. 

The dimensions of the grid box were 60 × 60 × 60 with 0.375 Å distance between points. Autodock4 and Lamarckian genetic algorithm was used to dock 250 conformations for each inhibitor (Molinspiration Database) [21]. Discovery studio 5.0 visualizer was used to investigate the enzyme-inhibitor non-bonding interactions.

### 2.9. Statistical Analysis

In vitro scavenging/reducing and enzyme inhibitory assays were means ± S.D. of three experiments conducted in triplicate. Data were analyzed by analysis of variance (ANOVA) coupled to Tukey post hoc test. Statistical significance was set at *p* < 0.05, and GraphPad Prism version 5.01 for Windows (GraphPad Software, San Diego, CA) was employed for the analysis. Regarding the ex vivo studies, the statistical analysis to calculate the number of animals used in each experiment (N = 4 per condition) was performed with the software G*Power (v3.1.9.4). Study potency (1-β) and significance level (α) were 0.8 and 0.05, respectively.

## 3. Results and Discussion

The water hemp extract was formerly analyzed from a phytochemical point of view. Total phenolics and flavonoids were reported in Table 1, whereas the phenolic fingerprint profile of the extract was detailed in our previous paper [10].

**Figure 1:**
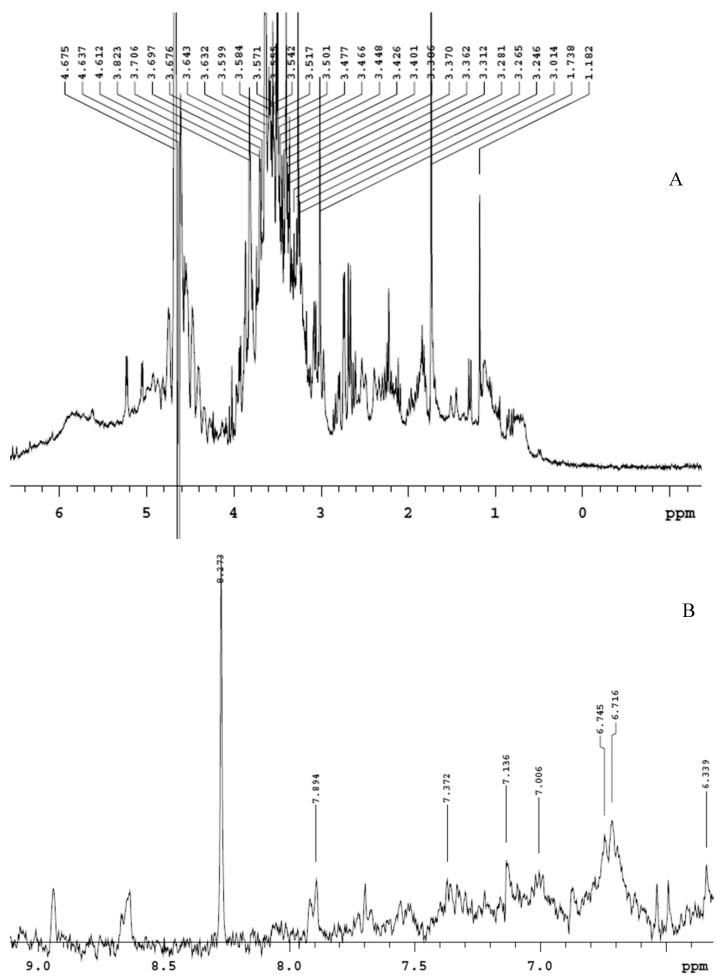
Qualitative composition of hemp water extract shown by ^1^H-NMR analysis with suppression of water signal. (**A**) In the chemical shift range 3–4, it is detected the presence of sugar fraction, whereas, in the chemical shift range 5–8 (**B**), the signals of phenolic compounds are shown.

The extract displayed a higher content of rutin, that was considered putatively responsible, at least in part, of the protective effects exerted in the colon [10]. The phenolic profile is also consistent with the scavenger, reducing, chelating and anti-tyrosinase properties (Table 1), thus suggesting intrinsic antioxidant and enzyme inhibition effects that could account for multiple pharmacological applications. Furthermore, the extract was also analyzed through ^1^H-NMR. The suppression of water signal was directly applied, in order to explore the qualitative composition of the water extract itself [22]. The chemical shift values, in the range 3–4 ppm (Figure 1A), indicate that sugar fraction represents most of the extract phytocomplex, whereas the chemical shift at 6–9 ppm (Figure 1B) is due to the presence of phenolic compounds [23,24]. The lower intensity of phenol signals in ^1^H-NMR spectra is consistent with limited phenolic compounds’ concentrations previously described [10].

**Figure 2:**
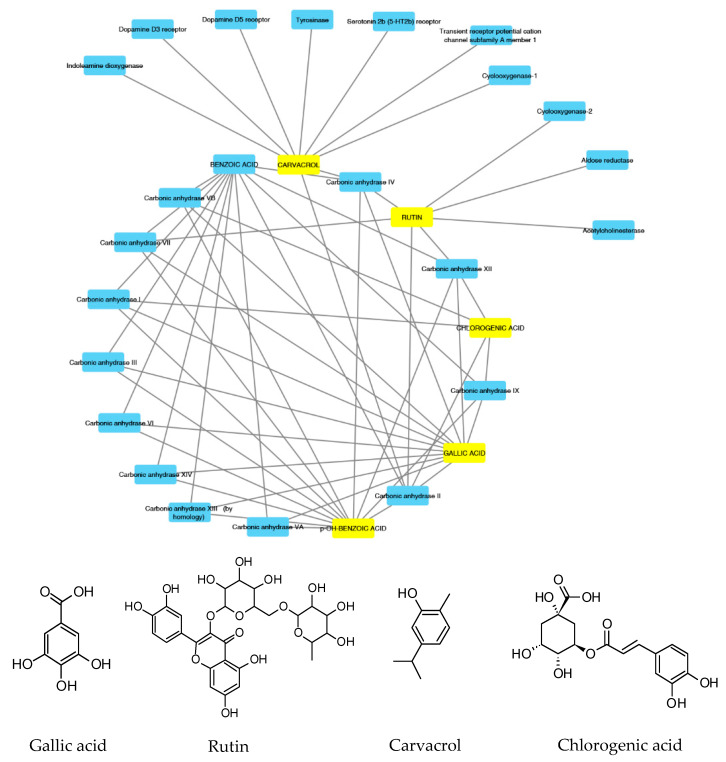
Components–Targets analysis. Compounds’ interactions with single predicted targets.

**Figure 3:**
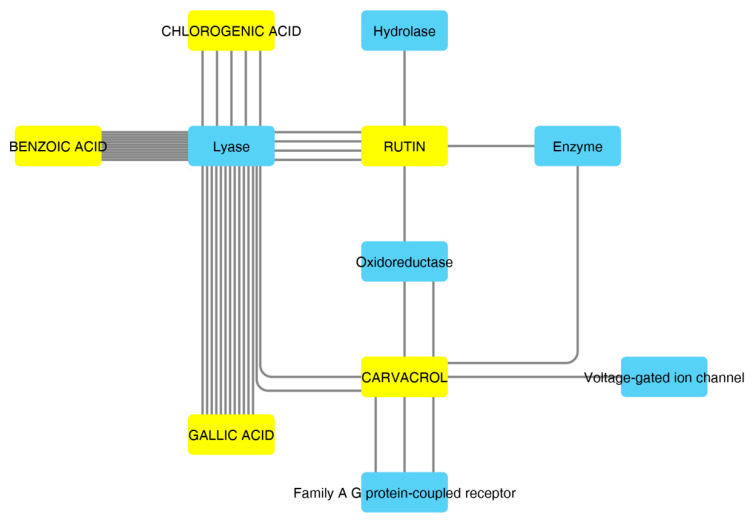
Components–Targets analysis. The predicted interactions between selected compounds and target protein classes are shown.

According to the aforementioned fingerprint analysis [10] that showed the presence of rutin, chlorogenic acid, gallic acid and carvacrol, a bioinformatics analysis was conducted in order to predict the pharmacological properties of the water extract, in terms of pharmacokinetics and putative protein targets, that were explored through the SwissADME software and SwissPredictionTarget software, respectively. Complete results are reported as Supplementary Material (Supplementary Material_Bioinformatics Folder), whereas the data about rutin, chlorogenic acid, gallic acid and carvacrol are depicted in Figure 2 and Figure 3.

These compounds were selected according to the best pharmacokinetics profile, that is the capability to cross biological membranes. Afterwards, the network pharmacology profile was built, plotting the selected compounds towards the putative targets yielded by SwissTagetPrediction software. The results of network pharmacology approach indicate the putative interaction of most selected phenolic compounds towards multiple carbonic anhydrase (CA) isoforms, and this was consistent with our recent findings [25]. Intriguingly, the sole carvacrol showed potential capability to interact with cyclooxygenase-1 (COX-1). Considering the previously observed efficacy of water hemp extract to blunt pro-inflammatory biomarkers [10], including prostaglandin production, ex vivo, and considering the up-regulated levels of both CA IX and COX-1 in colon cancer, thus suggesting the inhibition of these enzymes as a promising pharmacological tool to counteract colon cancer [26,27], we further investigated the putative interactions between gallic acid, chlorogenic acid and carvacrol towards these targets, through a docking approach.

**Figure 4:**
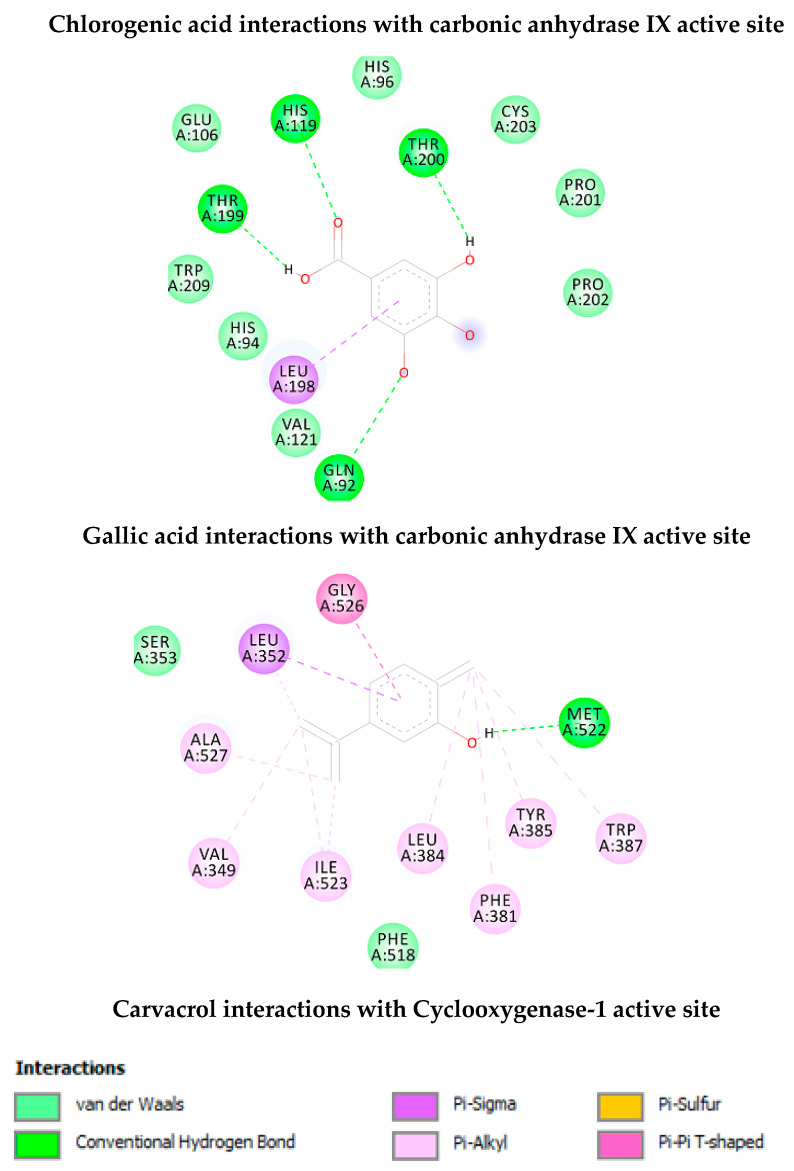
Non-bonding interactions of the docked compounds.

As shown in Table 2, chlorogenic acid has shown higher binding energy against carbonic anhydrase IX enzyme in comparison with gallic acid. Interestingly, the binding affinity of the chlorogenic acid was higher than the calculated binding affinity of the co-crystalized molecule (5-acetamido-1,3,4-thiadiazole-2-sulfonamide) (−6.65 kcal/mol). Obviously, the higher affinity of the chlorogenic acid is attributed to the strong interaction with enzyme active site through the formation of eight hydrogen bonds with the active site residues as shown in Figure 4. Similarly, the calculated binding affinity of the carvacrol against the cyclooxygenase-1 enzyme is shown in Table 2 and its non-bonding interactions in Figure 4.

**Figure 5:**
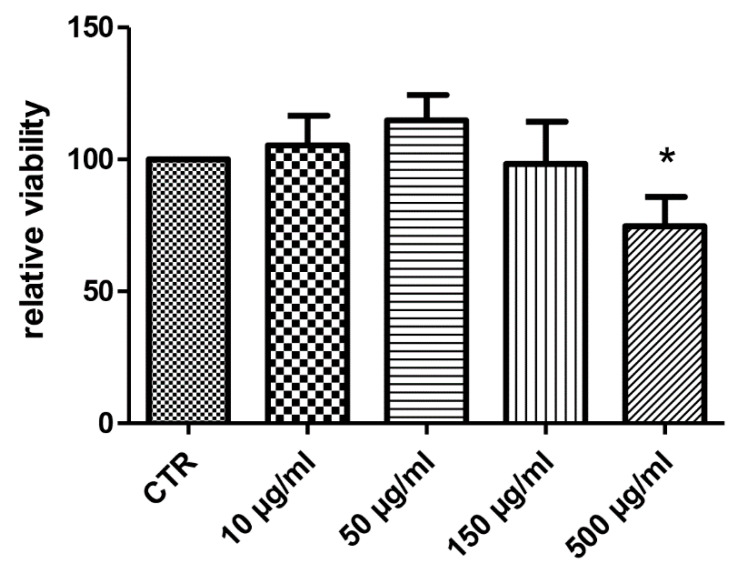
Anti-proliferative effect induced by hemp water extract (10–500 µg/mL) on human colon cancer HCT116 cell line. ANOVA, *p* < 0.05; * *p* < 0.05 vs. CTR group.

Considering the docking results on CA IX and COX-1 enzymes, the water extract was also investigated for evaluating anti-proliferative effects towards the human colon cancer HCT116 cell line. As also suggested by our previous study [10], the extract was tested in the concentration range 10–500 µg/mL. As depicted in Figure 5, the HCT116 cell line displayed a significant reduction of viability at the upper tested concentration (500 µg/mL), thus suggesting potential anti-proliferative effects. At the same concentration, the extract was also assayed in the wound healing assay, in order to explore a potential role on the spontaneous migration of HCT116 cells.

The sensitivity of HCT116 cells to the hemp water extract could be related to the intrinsic antioxidant/anti-inflammatory activity of the extract, that could disrupt the production of pro-inflammatory and anti-apoptotic factors stimulating cell proliferation [28].

**Figure 6:**
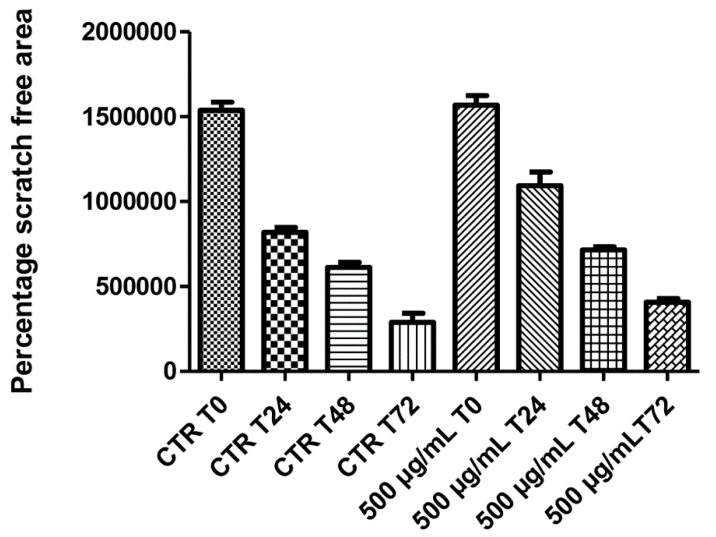
Null effect induced by hemp water extract (500 µg/mL) on the spontaneous migration (wound healing paradigm) of human colon cancer HCT116 cell line, at different time points (0, 24, 48, 72 h), following experimental lesion of cell monolayer.

On the other hand, literature data also suggest the stimulating effect of antioxidants on HCT116 cell viability [29], whereas the null effect observed on spontaneous migration (Figure 6) rules out any influence of this extract in modifying the intrinsic invasiveness capacity of this cancer cell line.

**Figure 7:**
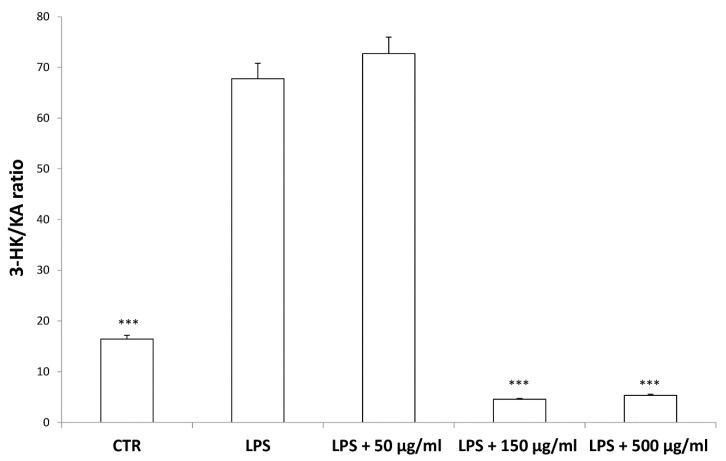
Inhibitory effects induced by hemp water extracts (50–500 µg/mL) on lipopolysaccharide (LPS)-induced 3-HK/KA ratio in isolated rat colon challenged with LPS. ANOVA, *p* < 0.0001; *** *p* < 0.001 vs. respective LPS group.

**Figure 8:**
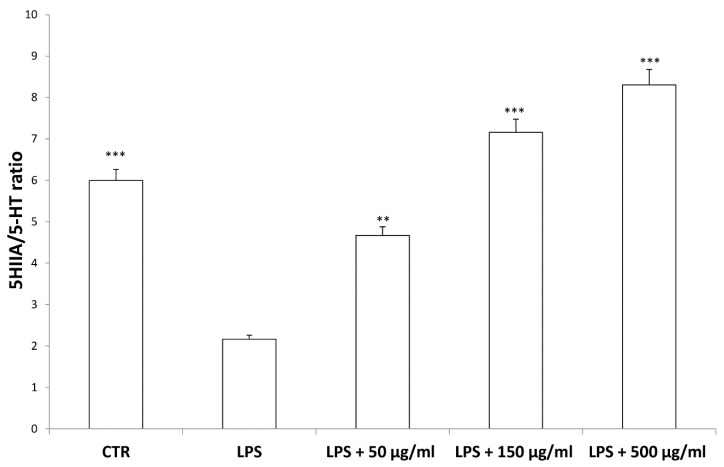
Inhibitory effects induced by hemp water extracts (50–500 µg/mL) on LPS-induced 5HIIA/5-HT ratio in isolated rat colon challenged with LPS. ANOVA, *p* < 0.0001; *** *p* < 0.001, ** *p* < 0.01 vs. respective LPS group.

According to bioinformatics and docking analyses, the observed anti-proliferative effect could depend on the putative inhibition of CA IX and COX-1 enzymes. On the other hand, the anti-serotoninergic effect displayed by this extract in the colon could be crucial [10]. In this regard, we should consider that serotonin, besides showing a pro-inflammatory effect, in the colon, could also exert mitogen effects, thus leading to increased cell viability, whereas rutin, that is reported to be the most representative flavonoid, in the extract, was suggested to counteract serotonin signaling [30]. Furthermore, the bioinformatics analysis demonstrated putative interactions of carvacrol with 5-HT2B receptor (Supplementary Material_Bioinformatics Folder), that was reported to mediate 5-HT excitatory effects in human colon, in vitro [31]. In our previous study [10], the blunting effect of water hemp extract was demonstrated against LPS-induced production of 5-HT, in rat colon. In the present study, we further explored the effects of hemp extract on 5-HT metabolism, taking into consideration the level of 5-HIIA, the main 5-HT metabolite and the levels of KA and 3-HK, that are the two main metabolites of kynurenine (KYN). KYN represents, together with 5-HT, one of the two major tryptophan degradative pathways [32]. In the colon, the challenging with a pro-inflammatory status is able to increase both the production of 5-HT [33], and the activity of kinurenine-3-monooxygenase (KMO) [34]. KMO is the kynurenine pathway-deriving enzyme involved in the production of 3-HK, especially in pro-inflammatory conditions, whereas kynurenine amino transferase (KAT) catalyzes the conversion of kynurenine in KA [35]. 3-HK and KA levels are inversely correlated in pro-inflammatory conditions, and in the present experimental paradigm the increased 3-HK/KA could be related, albeit partially, to the up-regulation of KMO [34]. In fact, the 3-HK/KA ratio could be also considered as an index of conversion of KYN in 3-HK [36], whereas the 5HIIA/5-HT represents a well-recognized index of 5-HT turnover [37]. In the present study, the LPS stimulus was not only effective in up-regulating 3-HK/KA (Figure 7), but also in decreasing 5HIIA/5-HT (Figure 8), thus suggesting a shift of tryptophan metabolism towards the production of pro-inflammatory metabolites, in the colon, whereas the water extract was able to counteract these effects. It is of noteworthy interest that the bioinformatics analysis predicted the potential interactions of carvacrol towards indoleamine 2,3-dioxygenase (IDO) (Supplementary Material_Bioinformatics Folder) that represents the rate limiting enzyme related to the tryptophan degradation, in the KYN pathway [38].

**Figure 9:**
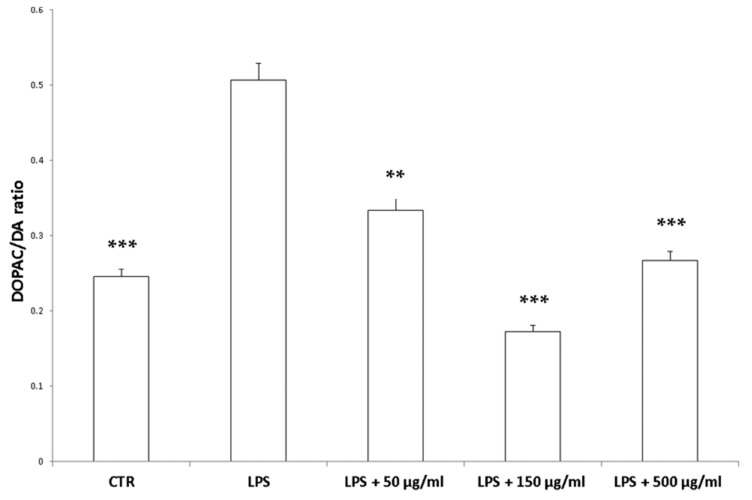
Inhibitory effects induced by hemp water extracts (50–500 µg/mL) on LPS-induced DOPAC/DA ratio in isolated rat colon challenged with LPS. ANOVA, *p* < 0.0001; *** *p* < 0.001, ** *p* < 0.01 vs. respective LPS group.

**Figure 10:**
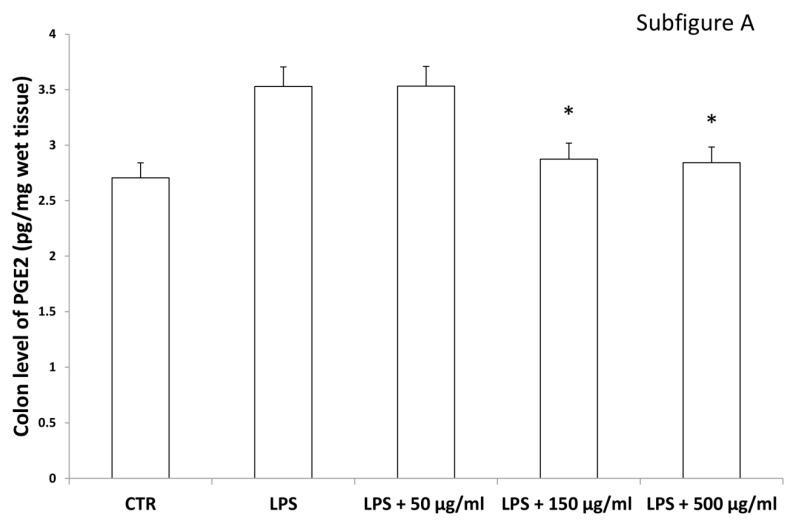

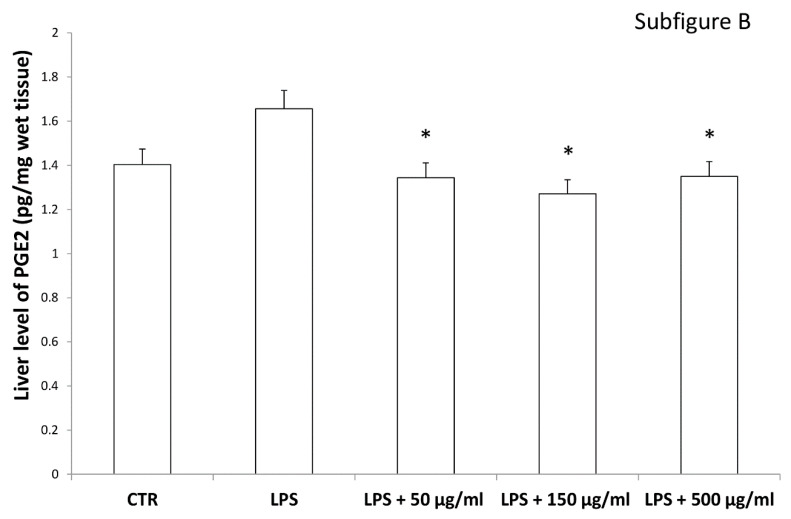
Inhibitory effects induced by hemp water extracts (50–500 µg/mL) on LPS-induced levels of prostaglandin (PG)E_2_ (pg/mg wet tissue) in isolated rat colon (**A**) and liver (**B**) challenged with LPS. ANOVA, *p* < 0.01; * *p* < 0.05 vs. respective LPS group.

Interestingly, in the brain, the increased 3-HK/KA ratio following pro-inflammatory conditions was also related to the IDO up-regulation [36]. The reduction of 3-HK/KA ratio following hemp water extract treatment is paralleled by an obvious and concomitant increase in the concentration of KA, that was also suggested to exert anti-proliferative effects in colon cancer [39], with regards to the HCT116 cell line, as well [28]. In this context, it is also sensitive to hypothesize that the anti-proliferative effects displayed by the present extract, against HCT116 cells, could be a consequence of the water extract-induced increase of KA production, in the colon. Regarding the 3-HK, it is also interesting to highlight that its production is increased in isolated liver challenged with LPS [40]. In the same paradigm, this increase was paralleled by a concomitant reduction of DA, whose turnover, measured as DOPAC/DA ratio [37], was also decreased by the hemp water extract, in the colon (Figure 9). DA, besides functioning as a key brain neurotransmitter, was also described as a regulator of immune/inflammatory response, in peripheral tissues [41]. Basically, about 50% DA is produced in the gastrointestinal tract, and delivered via portal vein to the liver, thus modulating the inflammatory response [42,43]. Overall, the capability of hemp water extract in contrasting the alterations induced by LPS on the synthesis and turnover of such metabolites, suggest anti-inflammatory effects that are consistent with the shown scavenger and reducing properties and confirmed by the reduction of colon and liver levels of PGE_2_ (Figure 10A,B), the main product of COX-2 that was suggested to be up-regulated in the present experimental paradigm [15]. We cannot exclude that the blunting effects on PGE_2_ could be related, at least in part, to the content of rutin. In this regard, bioinformatics analysis (Supplementary Material_Bioinformatics Folder) predicted the sole rutin as a candidate compound to interact with COX-2.

**Figure 11:**
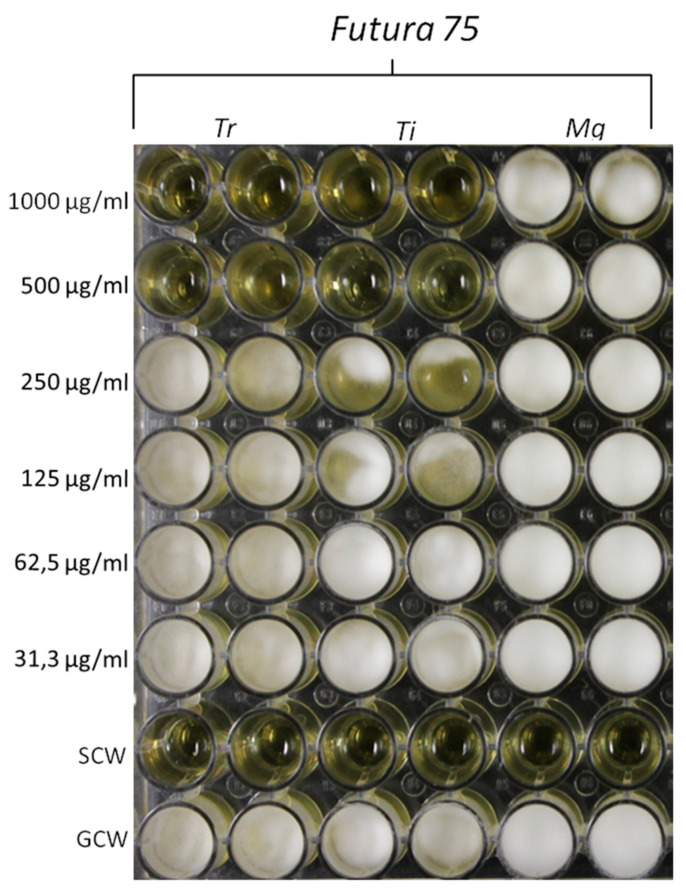
Inhibitory effects induced by Futura 75 water extract on the growth of *T. rubrum* (Tr) and *T. interdigitale* (Ti). The same extract displayed a null effect on the growth of *M. gypseum* (Mg). The inhibitory effects of the extract were qualitatively compared to growth control-well (GCW: containing medium, inoculum and the same amount of DMSO used in CTW, but compound-free), and sterility control well (SCW: sample, medium and sterile water instead of inoculum).

Based on the anti-mycotic effects showed by our previous investigation, especially towards *C. albicans* [10], in the present study we also evaluated the effects of the extract on different fungi strains, namely *T. rubrum*, *T. interdigitale* and *M. gypseum*, involved in multiple skin alterations, including infectious granuloma and hyperpigmentation [12,44,45]. The water extract of Futura 75 was active against *T. rubrum* and *T. interdigitale* with MICs of 500 µg/mL and 1000 µg/mL, respectively (Figure 11). No activity was observed against *M. gypseum.* These results could be added to the aforementioned inhibitory effect of the extract against tyrosinase (Table 1), whose inhibition represents a cornerstone in the management of skin hyperpigmentation [46].

**Figure 12:**
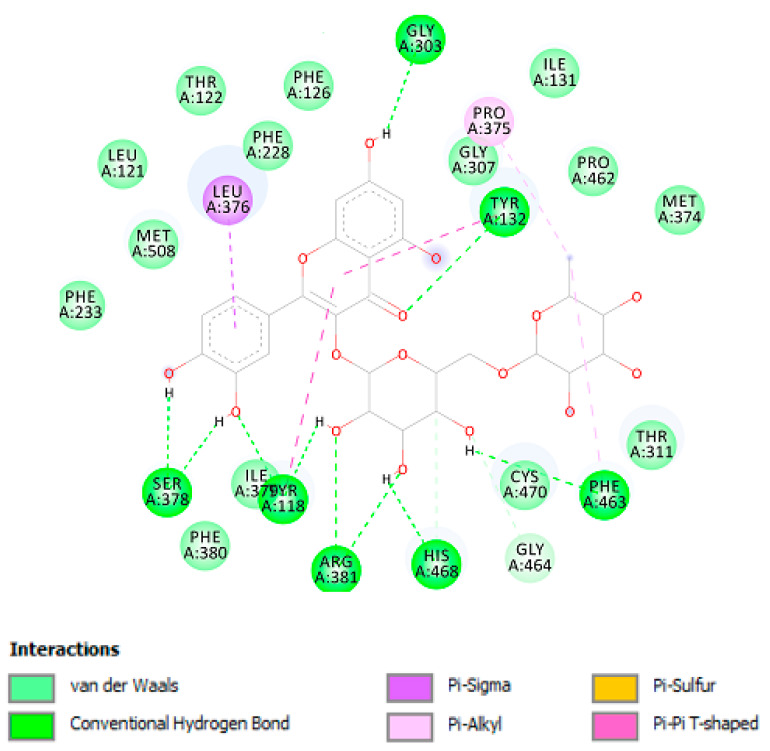
Rutin interactions with Lanosterol 14-α-demethylase active site; Non-bonding interactions of rutin with Lanosterol 14-α-demethylase enzyme.

Additionally, plant-derived compounds have long been considered as promising anti-tyrosinase and antimycotic agents [47,48]. According to the bioinformatics analysis (Supplementary Material_Bioinformatics Folder), carvacrol could be responsible of the observed enzyme inhibition against tyrosinase, whereas, according to the fingerprint analysis (Table 2), we hypothesize that carvacrol and rutin could mediate, at least in part, the observed antimycotic activity [49,50]. Intriguingly, the MIC values related to the antimycotic effects of rutin [49] are consistent with rutin concentration in the tested extract (Table 2). In this regard, a docking study was conducted to investigate potential interactions of rutin with lanosterol 14-α-demethylase (Figure 12), an enzyme involved in the ergosterol synthesis, in fungi, thus representing a key target of antimycotic therapy [51].

As shown in Table 3 and Figure 12, rutin has shown high affinity against lanosterol 14-α-demethylase enzyme. Due to the abundant hydroxyl groups in the structure of this compound, it has strong interactions with the enzyme active site with ten hydrogen bonds that may explain the high affinity of this compound.

Overall, these results further suggest the efficacy of water hemp extract in contrasting the clinical symptoms related to dermatophytes infection, including hyperpigmentation.

## 4. Conclusions

Concluding, the present findings highlight the potential of water extracts from industrial hemp inflorescences as sources of natural compounds that, besides the intrinsic antiradical activity, possess discrete mechanisms related to anti-inflammatory, anti-proliferative and antimycotic effects. In this context, and also in view of a more sustainable circular economy, it is desirable an improvement of industrial hemp chain production, taking into consideration the female inflorescences as high quality by-products with putative nutraceutical and cosmeceutical applications.

## Figures and Tables

**Table 1:** Total phenolic, flavonoid content, antioxidant parameters and tyrosinase inhibitory effect.

Parameters	Results
Total phenolic content (mg GAE/g)	21.16 ± 0.10
Total flavonoid content (mg RE/g)	7.05 ± 0.63
DPPH (mg TE/g)	14.87 ± 0.59
ABTS (mg TE/g)	39.00 ± 0.43
CUPRAC (mg TE/g)	47.53 ± 0.18
FRAP (mg TE/g)	27.53 ± 0.27
Tyrosinase (mg KAE/g)	18.67 ± 0.28

GAE: Gallic acid equivalents; RE: Rutin equivalents; TE: Trolox equivalents. KAE: Kojic acid equivalents. Values are reported as mean ± SD of three parallel experiments.

**Table 2:** The calculated binding free energy, ∆G, in kcal/mol, inhibition constant Ki, the key residues and the number of hydrogen atoms of the docked compounds.

Targets	∆G (K_i_)	Key Residues	No. of HB
**Carbonic Anhydrase IX**			
Chlorogenic Acid	−7.30 (4.5 µuM)	His64 (HB), Asn62 (HB), Thr199 (HB), His119 (HB), Glu106 (HB), Pro201 (HB), Trp5 (HB), Leu198, Thr200	8
Gallic Acid	−4.97 (228.8 µM)	Thr199 (HB), His119 (HB), Thr200 (HB), Gln92 (HB), Leu198	4
**Cyclooxygenase-1**			
Carvacrol	−6.03 (38.2 µM)	Met522 (HB), Trp387, Tyr385, Phe381, Leu384, Ile523, Val349, Leu352, Gly526	1

**Table 3:** The calculated binding free energy, ∆G, in kcal/mol, inhibition constant Ki, the key residues and the number of hydrogen atoms of the docked compounds.

Targets	∆G (K_i_)	Key Residues	No. of HB
Lanosterol 14-α-demethylase			
Rutin	−8.74 (390.0 nM)	Gly303 (HB), Tyr132 (HB), Phe463 (HB), His468 (HB), Arg381 (HB), Tyr118 (HB), Ser378 (HB), Leu376, Pro375.	10

## Supplementary Materials

The supplementary material about Bioinformatics are available online at https://www.mdpi.com/2076-3921/9/5/437/s1. Supplementary Material_Bioinformatics Folder

## References

[1] Montserrat-de la Paz S., Marín-Aguilar F., García-Gimenez M.D., Fernández-Arche M. (2014). Hemp (Cannabis sativa L.) seed oil: Analytical and phytochemical characterization of the unsaponifiable fraction. J. Agric. Food Chem..

[2] Vonapartis E., Aubin M.-P., Seguin P., Mustafa A.F., Charron J.-B. (2015). Seed composition of ten industrial hemp cultivars approved for production in Canada. J. Food Compos. Anal..

[3] Kiralan M., Gül V., Kara S.M. (2010). Fatty acid composition of hempseed oils from different locations in Turkey. Span. J. Agric. Res..

[4] De Backer B., Maebe K., Verstraete A.G., Charlier C. (2012). Evolution of the content of THC and other major cannabinoids in drug-type cannabis cuttings and seedlings during growth of plants. J. Forensic Sci..

[5] Amaducci S., Scordia D., Liu F., Zhang Q., Guo H., Testa G., Cosentino S. (2015). Key cultivation techniques for hemp in Europe and China. Ind. Crop Prod..

[6] Benelli G., Pavela R., Lupidi G., Nabissi M., Petrelli R., Kamte S.L.N., Cappellacci L., Fiorini D., Sut S., Dall’Acqua S. (2018). The crop-residue of fiber hemp cv. Futura 75: From a waste product to a source of botanical insecticides. Environ. Sci. Pollut. R..

[7] Smeriglio A., Trombetta D., Cornara L., Valussi M., De Feo V., Caputo L. (2019). Characterization and phytotoxicity assessment of essential oils from plant byproducts. Molecules.

[8] Zengin G., Menghini L., Di Sotto A., Mancinelli R., Sisto F., Carradori S., Cesa S., Fraschetti C., Filippi A., Angiolella L. (2018). Chromatographic analyses, in vitro biological activities, and cytotoxicity of cannabis sativa l. Essential oil: A multidisciplinary study. Molecules.

[9] Bertoli A., Tozzi S., Pistelli L., Angelini L.G. (2010). Fibre hemp inflorescences: From crop-residues to essential oil production. Ind. Crop Prod..

[10] Ferrante C., Recinella L., Ronci M., Menghini L., Brunetti L., Chiavaroli A., Leone S., Di Iorio L., Carradori S., Tirillini B. (2019). Multiple pharmacognostic characterization on hemp commercial cultivars: Focus on inflorescence water extract activity. Food Chem. Toxicol..

[11] Mazidi M., Taraghdari S.B., Rezaee P., Kamgar M., Jomezadeh M.R., Hasani O.A., Soukhtanloo M., Hosseini M., Gholamnezhad Z., Rakhshandeh H. (2014). The effect of hydroalcoholic extract of Cannabis Sativa on appetite hormone in rat. J. Compl. Integr. Med..

[12] El-Zawawy N.A., Ali S.S. (2016). Pyocyanin as anti-tyrosinase and anti tinea corporis: A novel treatment study. Microg. Pathog..

[13] Zengin G., Locatelli M., Stefanucci A., Macedonio G., Novellino E., Mirzaie S., Dvorácskó S., Carradori S., Brunetti L., Orlando G. (2017). Chemical characterization, antioxidant properties, anti-inflammatory activity, and enzyme inhibition of Ipomoea batatas L. leaf extracts. Int. J. Food Prop..

[14] Chiavaroli A., Recinella L., Ferrante C., Locatelli M., Macchione N., Zengin G., Leporini L., Leone S., Martinotti S., Brunetti L. (2017). Crocus sativus, Serenoa repens and Pinus massoniana extracts modulate inflammatory response in isolated rat prostate challenged with LPS. J. Boil. Regul. Homeost. Agents.

[15] Menghini L., Ferrante C., Leporini L., Recinella L., Chiavaroli A., Leone S., Pintore G., Vacca M., Orlando G., Brunetti L. (2016). An hydroalcoholic chamomile extract modulates inflammatory and immune response in HT29 cells and isolated rat colon. Phytother. Res..

[16] Brunetti L., Leone S., Orlando G., Ferrante C., Recinella L., Chiavaroli A., Di Nisio C., Shohreh R., Manippa F., Ricciuti A. (2014). Hypotensive effects of omentin-1 related to increased adiponectin and decreased interleukin-6 in intra-thoracic pericardial adipose tissue. Pharmacol. Rep..

[17] di Giacomo V., Chiavaroli A., Orlando G., Cataldi A., Rapino M., Di Valerio V., Leone S., Brunetti L., Menghini L., Recinella L. (2020). Neuroprotective and Neuromodulatory Effects Induced by Cannabidiol and Cannabigerol in Rat Hypo-E22 cells and Isolated Hypothalamus. Antioxidants.

[18] CLSI (2018). Reference Method for Broth Dilution Antifungal Susceptibility Testing of Yeasts. Approved Standard.

[19] Gu L., Lu J., Li Q., Wu N., Zhang L., Li H., Xing W., Zhang X. (2020). A network-based analysis of key pharmacological pathways of Andrographis paniculata acting on Alzheimer’s disease and experimental validation. J. Ethnopharmacol..

[20] Pedretti A., Villa L., Vistoli G. (2004). VEGA—An open platform to develop chemo-bio-informatics applications, using plug-in architecture and script programming. J. Comput. Aided Mol. Des..

[21] The Molinspiration Database. http://www.molinspiration.com.

[22] Politi M., Zloh M., Pintado M.E., Castro P.M., Heinrich M., Prieto J.M. (2009). Direct metabolic fingerprinting of commercial herbal tinctures by nuclear magnetic resonance spectroscopy and mass spectrometry. Phytochem. Analysis.

[23] Anastasiadi M., Zira A., Magiatis P., Haroutounian S.A., Skaltsounis A.L., Mikros E. (2009). 1H NMR-based metabonomics for the classification of Greek wines according to variety, region, and vintage. Comparison with HPLC data. J. Agric. Food Chem..

[24] Boffo E.F., Tavares L.A., Tobias A.C., Ferreira M.M., Ferreira A.G. (2012). Identification of components of Brazilian honey by 1H NMR and classification of its botanical origin by chemometric methods. LWT.

[25] Sinan K.I., Zengin G., Zheleva-Dimitrova D., Etienne O.K., Fawzi Mahomoodally M., Bouyahya A., Lobine D., Chiavaroli A., Ferrante C., Menghini L. (2020). Qualitative Phytochemical Fingerprint and Network Pharmacology Investigation of Achyranthes aspera Linn. Extracts. Molecules.

[26] Patrignani P., Sacco A., Sostres C., Bruno A., Dovizio M., Piazuelo E., Di Francesco L., Contursi A., Zucchelli M., Schiavone S. (2017). Low-dose aspirin acetylates cyclooxygenase-1 in human colorectal mucosa: Implications for the chemoprevention of colorectal cancer. Clin. Pharmacol. Ther..

[27] Shamsi F., Hasan P., Queen A., Hussain A., Khan P., Zeya B., King H.M., Rana S., Garrison J., Alajmi M.F. (2020). Synthesis and SAR studies of novel 1, 2, 4-oxadiazole-sulfonamide based compounds as potential anticancer agents for colorectal cancer therapy. Bioorg. Chem..

[28] Sinan K.I., Chiavaroli A., Orlando G., Bene K., Zengin G., Cziáky Z., Jekő J., Mahomoodally M.F., Picot-Allain M.C.N., Menghini L. (2020). Biopotential of bersama abyssinica fresen stem bark extracts: UHPLC profiles, antioxidant, enzyme inhibitory, and antiproliferative propensities. Antioxidants.

[29] Salah A., Bouaziz C., Amara I., Abid-Essefi S., Bacha H. (2019). Eugenol protects against citrinin-induced cytotoxicity and oxidative damages in cultured human colorectal HCT116 cells. Environ. Sci. Pollut. R..

[30] Chen W., Jin M., Wu W. (2002). Experimental study on inhibitory effect of rutin against platelet activation induced by platelet activating factor in rabbits. Zhongguo Zhong Xi Yi Jie He Za Zhi.

[31] Borman R., Tilford N., Harmer D., Day N., Ellis E., Sheldrick R., Carey J., Coleman R., Baxter G. (2002). 5-HT2B receptors play a key role in mediating the excitatory effects of 5-HT in human colon in vitro. Br. J. Pharmacol..

[32] Dolivo D.M., Larson S.A., Dominko T. (2018). Tryptophan metabolites kynurenine and serotonin regulate fibroblast activation and fibrosis. Cell. Mol. Life Sci..

[33] Stavely R., Fraser S., Sharma S., Rahman A.A., Stojanovska V., Sakkal S., Apostolopoulos V., Bertrand P., Nurgali K. (2018). The onset and progression of chronic colitis parallels increased mucosal serotonin release via enterochromaffin cell hyperplasia and downregulation of the serotonin reuptake transporter. Inflamm. Bowel Dis..

[34] Tashita C., Hoshi M., Hirata A., Nakamoto K., Ando T., Hattori T., Yamamoto Y., Tezuka H., Tomita H., Hara A. (2020). Kynurenine plays an immunosuppressive role in 2, 4, 6-trinitrobenzene sulfate-induced colitis in mice. World J. Gastroenterol..

[35] Maddison D.C., Giorgini F. (2015). The kynurenine pathway and neurodegenerative disease. Seminars in Cell & Developmental Biology.

[36] Jiang X., Xu L., Tang L., Liu F., Chen Z., Zhang J., Chen L., Pang C., Yu X. (2018). Role of the indoleamine-2, 3-dioxygenase/kynurenine pathway of tryptophan metabolism in behavioral alterations in a hepatic encephalopathy rat model. J. Neuroinflammion.

[37] Lee J., Chang C., Liu I., Chi T., Yu H., Cheng J. (2001). Changes in endogenous monoamines in aged rats. Clin. Exp. Pharmacol. Physiol..

[38] Zara C., Severino A., Flego D., Ruggio A., Pedicino D., Giglio A.F., Trotta F., Lucci C., D’Amario D., Vinci R. (2018). Indoleamine 2, 3-dioxygenase (IDO) enzyme links innate immunity and altered T-cell differentiation in non-ST segment elevation acute coronary syndrome. Int. J. Mol. Sci..

[39] Walczak K., Turski W.A., Rajtar G. (2014). Kynurenic acid inhibits colon cancer proliferation in vitro: Effects on signaling pathways. Amino Acids.

[40] Mahomoodally M.F., Sinan K.I., Bene K., Zengin G., Orlando G., Menghini L., Veschi S., Chiavaroli A., Recinella L., Brunetti L. (2020). Bridelia speciosa Müll. Arg. Stem bark Extracts as a Potential Biomedicine: From Tropical Western Africa to the Pharmacy Shelf. Antioxidants.

[41] Zhou H., Tang L., Yang Y., Lin L., Dai J., Ge P., Ai Q., Jiang R., Zhang L. (2018). Dopamine alleviated acute liver injury induced by lipopolysaccharide/D-galactosamine in mice. Int. Immunopharmacol..

[42] Eisenhofer G., Åneman A., Friberg P., Hooper D., Fåndriks L., Lonroth H., Hunyady B.I., Mezey E. (1997). Substantial production of dopamine in the human gastrointestinal tract. J. Clin. Endocrinol. Metab..

[43] Xue R., Zhang H., Pan J., Du Z., Zhou W., Zhang Z., Tian Z., Zhou R., Bai L. (2018). Peripheral dopamine controlled by gut microbes inhibits invariant natural killer T cell-mediated hepatitis. Front. Immunol..

[44] Isa-Isa R., Arenas R., Isa M. (2010). Inflammatory tinea capitis: Kerion, dermatophytic granuloma, and mycetoma. Clin. Dermatol..

[45] Zhang M., Jiang L., Li F., Xu Y., Lv S., Wang B. (2019). Simultaneous dermatophytosis and keratomycosis caused by Trichophyton interdigitale infection: A case report and literature review. BMC Infect. Dis..

[46] Kolbe L., Mann T., Gerwat W., Batzer J., Ahlheit S., Scherner C., Wenck H., Stäb F. (2013). 4-n-butylresorcinol, a highly effective tyrosinase inhibitor for the topical treatment of hyperpigmentation. J. Eur. Acad. Dermatol..

[47] Bottari N.B., Lopes L.Q.S., Pizzuti K., dos Santos Alves C.F., Corrêa M.S., Bolzan L.P., Zago A., de Almeida Vaucher R., Boligon A.A., Giongo J.L. (2017). Antimicrobial activity and phytochemical characterization of Carya illinoensis. Microb. Pathog..

[48] Sarikurkcu C., Locatelli M., Mocan A., Zengin G., Kirkan B. (2019). Phenolic Profile and Bioactivities of Sideritis perfoliata L.: The Plant, Its Most Active Extract, and Its Broad Biological Properties. Front. Pharmacol..

[49] Orhan D.D., Özçelik B., Özgen S., Ergun F. (2010). Antibacterial, antifungal, and antiviral activities of some flavonoids. Microbiol. Res..

[50] Shaban S., Patel M., Ahmad A. (2020). Improved efficacy of antifungal drugs in combination with monoterpene phenols against Candida auris. Sci. Rep..

[51] Houšť J., Spížek J., Havlíček V. (2020). Antifungal Drugs. Metabolites.

